# Pan-cancer Analysis Reveals m^6^A Variation and Cell-specific Regulatory Network in Different Cancer Types

**DOI:** 10.1093/gpbjnl/qzae052

**Published:** 2024-07-05

**Authors:** Yao Lin, Jingyi Li, Shuaiyi Liang, Yaxin Chen, Yueqi Li, Yixian Cun, Lei Tian, Yuanli Zhou, Yitong Chen, Jiemei Chu, Hubin Chen, Qiang Luo, Ruili Zheng, Gang Wang, Hao Liang, Ping Cui, Sanqi An

**Affiliations:** Life Sciences Institute, Biosafety Level-3 Laboratory, Guangxi Medical University, Nanning 530021, China; Life Sciences Institute, Biosafety Level-3 Laboratory, Guangxi Medical University, Nanning 530021, China; Department of Pathology, Guangdong Second Provincial General Hospital, Guangzhou 510317, China; Department of Bioinformatics, Anjin Biotechnology Co., Ltd., Guangzhou 510000, China; Frontiers Science Center for Disease-related Molecular Network, Precision Medicine Research Center, West China Hospital, Department of Respiratory and Critical Care Medicine, Sichuan University, Chengdu 610041, China; School of Basic Medical Sciences, Guangxi Medical University, Nanning 530021, China; Department of Medical Bioinformatics, Zhongshan School of Medicine, Sun Yat-sen University, Guangzhou 510080, China; The First Affiliated Hospital of Guangxi Medical University, Nanning 530021, China; The First Affiliated Hospital of Guangxi Medical University, Nanning 530021, China; The First Affiliated Hospital of Guangxi Medical University, Nanning 530021, China; Life Sciences Institute, Biosafety Level-3 Laboratory, Guangxi Medical University, Nanning 530021, China; Life Sciences Institute, Biosafety Level-3 Laboratory, Guangxi Medical University, Nanning 530021, China; Life Sciences Institute, Biosafety Level-3 Laboratory, Guangxi Medical University, Nanning 530021, China; Life Sciences Institute, Biosafety Level-3 Laboratory, Guangxi Medical University, Nanning 530021, China; Life Sciences Institute, Biosafety Level-3 Laboratory, Guangxi Medical University, Nanning 530021, China; Life Sciences Institute, Biosafety Level-3 Laboratory, Guangxi Medical University, Nanning 530021, China; Life Sciences Institute, Biosafety Level-3 Laboratory, Guangxi Medical University, Nanning 530021, China; Life Sciences Institute, Biosafety Level-3 Laboratory, Guangxi Medical University, Nanning 530021, China; School of Basic Medical Sciences, Guangxi Medical University, Nanning 530021, China

**Keywords:** *N* ^6^-methyladenosine, Heterogeneity, m^6^A-regulated gene, m^6^A regulator, Cell-specific

## Abstract

As the most abundant messenger RNA (mRNA) modification, *N*^6^-methyladenosine (m^6^A) plays a crucial role in RNA fate, impacting cellular and physiological processes in various tumor types. However, our understanding of the role of the m^6^A methylome in tumor heterogeneity remains limited. Herein, we collected and analyzed m^6^A methylomes across nine human tissues from 97 m^6^A sequencing (m^6^A-seq) and RNA sequencing (RNA-seq) samples. Our findings demonstrate that m^6^A exhibits different heterogeneity in most tumor tissues compared to normal tissues, which contributes to the diverse clinical outcomes in different cancer types. We also found that the cancer type-specific m^6^A level regulated the expression of different cancer-related genes in distinct cancer types. Utilizing a novel and reliable method called “*m^6^A-express*”, we predicted m^6^A-regulated genes and revealed that cancer type-specific m^6^A-regulated genes contributed to the prognosis, tumor origin, and infiltration level of immune cells in diverse patient populations. Furthermore, we identified cell-specific m^6^A regulators that regulate cancer-specific m^6^A and constructed a regulatory network. Experimental validation was performed, confirming that the cell-specific m^6^A regulator CAPRIN1 controls the m^6^A level of *TP53*. Overall, our work reveals the clinical relevance of m^6^A in various tumor tissues and explains how such heterogeneity is established. These results further suggest the potential of m^6^A in cancer precision medicine for patients with different cancer types.

## Introduction

It is widely accepted that cancer is a disease caused by genomic and epigenetic changes in oncogenes and tumor suppressor genes. Pan-cancer analysis of whole genomes, enhancer expression, long non-coding RNA (lncRNA) regulation, immune response and the like, aiming to examine the differences and similarities across different tumor types, has received extensive attention [1–4]. As the most prevalent messenger RNA (mRNA) modification, *N*^6^-methyladenosine (m^6^A) is reversibly regulated by various classical m^6^A regulators, including methyltransferases (METTL3, METTL14, VIRMA, ZC3H13, WTAP, CBLL1/HAKAI, and RBM15/RBM15B), which mediate methylation, and m^6^A demethylases (ALKBH5 and FTO), which mediate demethylation of different m^6^A sites [5–7]. m^6^A “readers”, YTH family proteins and IGF2BPs, recognize this reversible m^6^A and regulate post-transcriptional processes, such as RNA decay, alternative polyadenylation, and nuclear export, in different cancer types [8,9]. Previous studies have shown that m^6^A plays an important role in the progression of various cancer types [10,11]. However, our understanding of the mechanisms underlying cell type- and cancer type-specific m^6^A regulation in pan-cancer is limited [12,13].

To investigate the complex cell type-specific regulatory network and the role of m^6^A in different cancer types, it is necessary to perform a pan-cancer analysis at the level of m^6^A methylation. Due to the limitations of m^6^A identification methods, some researchers have only tried to perform gene expression analysis of classical m^6^A regulators to analyze the heterogeneity of m^6^A regulators instead of real m^6^A in different cancers [14,15]. However, key m^6^A regulators, such as METTL3, METTL14 and YTHDC2, may function independently of m^6^A [16–18]. Therefore, these m^6^A regulator expression-based pan-cancer analyses are far from revealing the characteristics and roles of m^6^A in different cancer types. Among the various methods for identifying m^6^A sites with high resolution, m^6^A sequencing (m^6^A-seq) is the most widely used and has promoted m^6^A research [19]. In our previous study, we made multiple methodological improvements to mitigate the impact of technical biases caused by different immunoprecipitation (IP) efficiencies across the different libraries in m^6^A-seq [13] to provide a reliable method for m^6^A pan-cancer analysis.

With accurate calculations based on a large number of m^6^A-seq datasets, we attempted to investigate the m^6^A landscape in terms of cancer tissue specificity. Then, we wanted to know what key clinically relevant heterogeneity of m^6^A leads to and how this heterogeneity of m^6^A is established. To achieve this, we performed comprehensive pan-cancer analyses involving nine cancer types utilizing 97 m^6^A-seq and RNA sequencing (RNA-seq) samples. We used a reliable method called “*m^6^A-express*” to predict m^6^A-regulated genes in pan-cancer analysis [12]. Through these m^6^A-regulated genes, we comparably and comprehensively explored the clinically relevant m^6^A modifications that are involved in cancer progression, prognostic prediction, and molecular classification across 31 different cancer types. Additionally, we attempted experimental validation of the CAPRIN1/METTL3–m^6^A–*TP53* axis identified by the *in silico* analysis. To the best of our knowledge, this is the first pan-cancer analysis based on global m^6^A levels. Our results will contribute to a better understanding of m^6^A heterogeneity and its role in cancer precision therapy.

## Results

### Systematic analysis of extensive m^6^A-seq data reveals distinct m^6^A features in nine types of cancer and normal tissues

We collected m^6^A-seq data of 97 tumor tissues as well as corresponding normal tissues and explored the characteristics of these m^6^A sites (Table S1). Various technical issues associated with m^6^A-seq data can obstruct the successful systematic analysis of quantitative m^6^A ratios calculated from m^6^A-seq data. As a result, we implemented multiple processing steps to minimize the effects of different types of technical issues (see the “Materials and methods” section for details). Due to technical biases in preparing the m^6^A-seq libraries, such as variations in RNA length, the shifting of peak centers and divergence of peak widths will be controlled using winscore method (see the “Materials and methods” section for details). Afterward, quantile normalization of the m^6^A ratios is performed to correct biases introduced by differences in antibody efficiencies across various laboratories. Based on our previous findings that m^6^A sites with coefficient of variation (CV) greater than 0.3 vary between different cells [13], we further calculated the specificity and function of these m^6^A sites. Subsequently, we utilized the expression levels of cell-specific m^6^A regulators identified in our previous research and classical m^6^A regulators to compute their correlation with the m^6^A levels we obtained. Through experimental validation, we identified specific regulators that modulate cancer-specific m^6^A. At the same time, we employed *m^6^A-express* to calculate target genes regulated by m^6^A in different cancers and analyzed the potential impact of these m^6^A-regulated genes on prognosis and cellular immune responses across various cancers (Figure S1).

**Figure 1:**
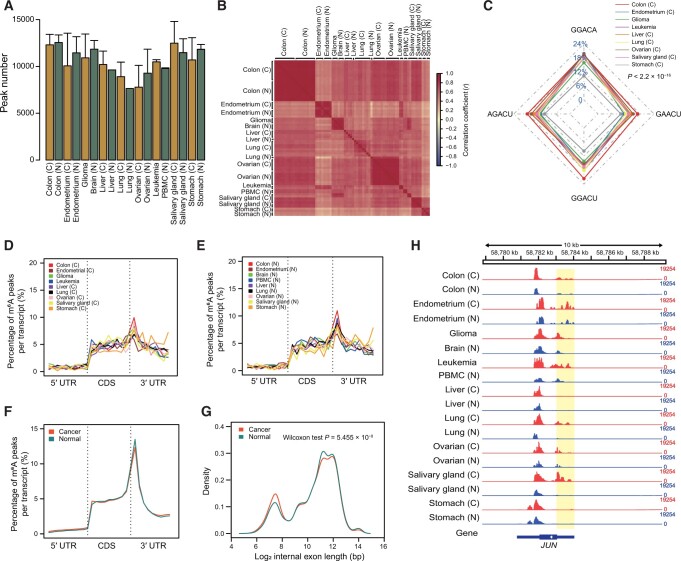
m^6^A features in nine cancer and normal tissues **A**. Number of m^6^A peaks identified in each cancer and normal tissue. Error bars denote the SD across all replicates. **B**. Pearson correlation heatmap showing the correlations of global m^6^A levels between different samples. **C**. The radar map showing the percentage of m^6^A sub-motifs of nine cancer tissues, indicated by different colored lines (Chi-squared test, *P* < 2.2 × 10^−16^). **D**. and **E**. Normalized distribution of the m^6^A peaks at 5′ UTR, CDS, and 3′ UTR in nine cancer tissues (D) and normal tissues (E). **F**. Comparison of overall m^6^A peak distribution between cancer and normal tissues. **G**. Comparison of m^6^A distribution along exon length between cancer and normal tissues. *P* value was determined by the Wilcoxon test. **H**. Tracks showing m^6^A coverage of the *JUN* gene from randomly selected samples among 97 cancer and normal subjects. The 5′ UTR was highlighted by yellow background. The data range for each track is displayed on the right side (0–19,254). N, normal; C, cancer; m^6^A, *N*^6^-methyladenosine; SD, standard deviation; UTR, untranslated region; CDS, coding sequence; PBMC, peripheral blood mononuclear cell.

To systematically reveal the basic characteristics of m^6^A in various cancer types at the tissue level, we initially made several methodological improvements to the winscore-based method to mitigate the impacts of technical biases of m^6^A-seq data from different labs (see the “Materials and methods” section for details) [13]. This is important for the reliable quantification of m^6^A levels using m^6^A-seq data from different labs. After performing quantile normalization of the m^6^A level across all tissues, we performed unsupervised clustering using the normalized m^6^A level and found that the variable m^6^A levels in lung cancer and leukemia were not clustered according to the labs but rather according to cancer types, suggesting that we successfully eliminate batch effects from different labs (Figure S2A). Then, we conducted m^6^A quantitative analysis on nine tumor tissues and their controls, including glioma, lung cancer, liver cancer, endometrial cancer, ovarian cancer, leukemia, colon cancer, salivary gland cancer, and stomach cancer. Approximately 10,000 m^6^A peaks were obtained for each tissue (**Figure 1**A), and samples from the same tissue type clustered well according to their m^6^A levels (Figure 1B), indicating that tissue-specific m^6^A methylomes indeed exist. To better understand the biological function of these m^6^A sites, we annotated them and found that m^6^A mostly occurred on protein-encoding genes (Figure S2B). Some m^6^A modifications were present on non-coding RNAs (ncRNAs; Figure S2B), suggesting that ncRNAs also play essential roles in these cancer types. We observed a trend that m^6^A from different cancer types had varying levels of enrichment in distinct m^6^A sub-motifs (*P* < 2.2 × 10^−16^) (Figure 1C, Figure S2C), and there were differences in the enrichment of m^6^A motifs in different tumor types (Figure S2D), suggesting that m^6^A methylomes may be cancer-specific. Cancer-specific m^6^A motifs, m^6^A distribution, and m^6^A peak number indicate that m^6^A with different functions in different tumors is regulated in a cancer-specific manner.

Then, we studied the distributions of m^6^A in tumor and normal samples, respectively (Figure 1D and E). Consistent with other reports, m^6^A was more likely to be enriched in the 3′ untranslated region (UTR) start segment and the coding sequence (CDS) segment and reached its peak at the 3′ UTR start segment in both tumor and normal samples. By integrating the distributions of m^6^A in tumor and normal samples, we found that in the start codon regions, m^6^A peaks were more prevalent in tumor samples than those in normal samples, while in the stop codon regions, m^6^A peaks in tumor samples were less abundant than those in normal tissues (Figure 1F). Moreover, m^6^A peaks in tumor samples were less abundant in long internal exons (*P* = 5.455 × 10^−8^) (Figure 1G). In our previous research, the m^6^A sites away from stop codons were as “dynamic m^6^A sites”, which were precisely and dynamically regulated by cell-specific *trans*-regulators expressed with spatial and temporal specificities across different cell types [13]. Herein, we suspected that m^6^A in cancer tissues had a greater CV than that of normal tissues, which might be regulated by cell-specific *trans*-regulators and contribute to the heterogeneity of cancer. We also used *JUN*, which is associated with human cancer malignancies, as an example to analyze the differences in the CV of m^6^A between cancer and normal tissues. At the beginning of the *JUN* gene coding region, higher CVs on the start codons were observed in nine types of cancer tissues (Figure 1H).

To further validate our conclusions, we also gathered m^6^A-seq data from cancer and normal cell lines for further analysis, including seven tumor cell lines (HEC-1-A, HepG2, iSLK, MOLM-13, MonoMac6, MT-4 T-cell, and NB4) and three normal cell lines (MSC, NHDF, and TIME) (Table S2). By analyzing the distribution and motif characteristics of m^6^A sites, we found that m^6^A in normal cell lines tended to be closer to stop codons, whereas there were more m^6^A peaks in cancer cell lines in coding regions (Figure S2E). Overall, the analysis results of m^6^A-seq data from cancer cell lines are consistent with those from tumor tissues, further indicating the reliability of our findings in tumor tissues.

### m^6^A shows different heterogeneity in most tumor tissues compared with normal tissues in human

**Figure 2:**
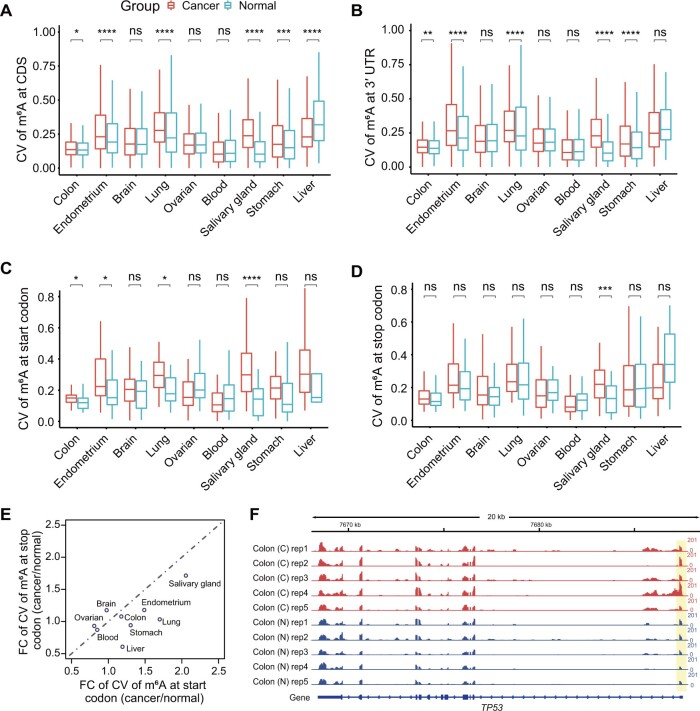
CV of the m^6^A level in nine cancer and normal tissues **A**.–**D**. Box plots showing CV of the m^6^A level at the CDS (A), 3′ UTR (B), start codon (C), and stop codon (D) segments. *P* values were determined by the Wilcoxon test (*, *P* < 0.05; **, *P* < 0.01; ***, *P* < 0.001; ****, *P* < 0.0001; ns, no significance). **E**. Dot plot representing FC of CV of m^6^A (cancer/normal) at the start codons and stop codons across nine tissue samples. **F**. Tracks displaying the m^6^A abundance of the *TP53* gene in a lung tumor. The 5′ UTR was highlighted by yellow background. The data range for each track is displayed on the right side (0–201). CV, coefficient of variation; FC, fold change.

The CVs of m^6^A were calculated in different regions of mRNA to explore whether the CVs in different cancer samples were greater than those in normal tissues. Variable peaks ranged from 15% to 50% in normal tissues (Figure S3A), consistent with a recent report that *cis*-regulation accounts for 33%–46% of the variability in m^6^A levels [20]. Moreover, the m^6^A peaks located far from stop codons had significantly higher CVs in colon cancer, endometrial cancer, lung cancer, salivary gland cancer, and stomach cancer (**Figure 2**A–D, Figure S3B). Therefore, there were more variable peaks in these cancer samples than in normal samples, suggesting that m^6^A in these five cancer types tends to be variable. We also calculated the m^6^A CV fold change (FC) of cancer with respect to normal tissues in start and stop codons. Figure 2E shows that m^6^A enriched in start codons tends to have higher CVs in cancer tissues than m^6^A in stop codons. Moreover, we used the *TP53* and *HSPD1* genes as examples to study variations in m^6^A in cancer, and found that *TP53* and *HSPD1* showed higher variations in m^6^A at the start of CDS in colon cancer and endometrial cancer than in normal tissues (Figure 2F, Figure S3C).

In addition to heterogeneity, we explored whether m^6^A in different cancer types has homogeneity. The samples were divided into two groups: the cancer group (45 samples) and the normal group (39 samples). We performed whole-genome differential m^6^A analysis between these two groups. Only 428 m^6^A sites were differentially methylated [false discovery rate (FDR) < 0.05] between pan-cancer and normal tissues. m^6^A at these sites showed higher heterogeneity (*P* < 2.2 × 10^−16^) (Figure S3D). Thus, our findings indicate that m^6^A displays different heterogeneity across different tumor tissues.

### Cancer type-specific m^6^A affects the expression of distinct cancer-related genes and immune-related genes

**Figure 3:**
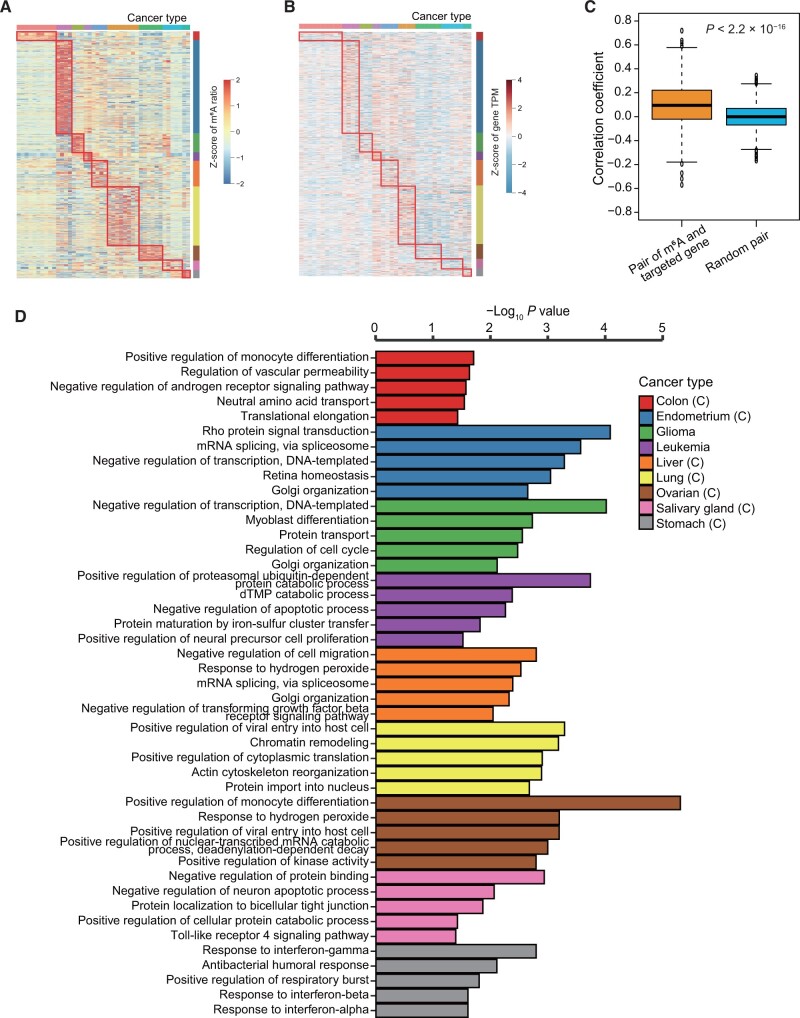
Correlation and impact of m^6^A methylation levels on gene expression in different cancer types **A**. Heatmap showing the m^6^A levels in nine cancer types. Each red box represents the cancer type-specific m^6^A sites of each cancer. **B**. Heatmap showing the expression levels of m^6^A-targeted genes after log_2_ conversion to TPM in nine cancer types. Each red box represents the cancer type-specific m^6^A-targeted genes of each cancer. **C**. The correlation between the level of m^6^A modification in each profile and the expression of m^6^A-targeted genes. The trend significantly differs from the results of randomly shuffled data for this correlation (*P* < 2.2 × 10^−16^). **D**. GO analysis of cancer-specific m^6^A-targeted genes in each cancer type. GO, Gene Ontology; TPM, transcripts per kilobase of exon model per million mapped reads.

To delve deeper into the role of cancer-specific m^6^A across various cancers, we identified m^6^A sites with a CV greater than 0.3 as cancer-specific in tumor samples. This is because we observed that m^6^A sites with a CV greater than 0.3 often exhibit cell-specific characteristics, as previously noted [13]. Clustering showed that m^6^A levels were strongly cancer type-specific (**Figure 3**A). We also performed gene expression analysis of m^6^A-targeted genes across all nine tumor tissues and found that gene expression may be correlated with m^6^A modification levels (Figure 3A and B). To further investigate the relationship between m^6^A and gene expression, we analyzed the correlation between the level of m^6^A modification at each site and the expression of the targeted gene (Figure 3C). We observed that the majority of m^6^A modifications exhibited a positive correlation with the expression of the targeted genes. This trend significantly differs from the results of randomly shuffled data for this correlation (*P* < 2.2 × 10^−16^). This outcome indicates that m^6^A modifications may be positively associated with gene regulation in tumor samples. This conclusion provides a valuable reference for guiding subsequent experimental research.

Gene set variation analysis (GSVA) was employed to explore the biological functions among these distinct m^6^A modification patterns, and the results showed that different cancer-specific m^6^A was involved in different regulatory pathways, including various immune-related pathways, highlighting the relevance of m^6^A and tumor immunity (Figure S4). For example, colon cancer-specific m^6^A was enriched in pathways such as natural killer cell mediated cytotoxicity, cytokine–cytokine receptor interaction, JAK/STAT signaling pathway, and Notch signaling pathway. Endometrial cancer-specific m^6^A was involved in B-cell receptor signaling pathway, T-cell receptor signaling pathway, and chemokine signaling pathway. Salivary gland cancer-specific m^6^A was associated with epithelial cell signaling in *Helicobacter pylori* infection and Toll-like receptor signaling pathway. Ovarian cancer-specific m^6^A was involved in autoimmune thyroid disease. In addition, in some cases, cancer-specific m^6^A was tissue-specific. For example, glioma-specific m^6^A was essential in neurotrophin signaling pathway and transforming growth factor (TGF) beta signaling pathway. Leukemia-specific m^6^A was related to acute myeloid leukemia and cell cycle pathway. We also identified cancer-specific m^6^A that was significantly involved in the regulation of key tumor pathways. For instance, liver cancer-specific m^6^A was related to the Wnt signaling pathway, lung cancer-specific m^6^A was enriched in glycerophospholipid metabolism, and stomach cancer-related m^6^A was involved in oxidative phosphorylation. These results illustrate the wide and varied regulatory mechanisms of m^6^A in different tumors.

We also performed Gene Ontology (GO) enrichment analysis (Figure 3D, Figure S5A and B). The analysis revealed significant enrichment of m^6^A in metabolic pathways, such as pyrimidine and purine and drug metabolism, as well as immune related pathways, including mitogen-activated protein kinase (MAPK) signaling, T-cell and B-cell pathways, leukocyte migration, infection, and inflammation. Analysis of cellular components showed that in pan-cancer, m^6^A was involved in the cell nucleus, cell membrane, Golgi apparatus, mitochondria, cytoplasm, endoplasmic reticulum, and exosome. These results are consistent with previous reports that m^6^A regulates tumor immunity and key tumor pathways [10]. Furthermore, these findings revealed that cancer-specific m^6^A tended to be enriched in different pathways and functions, highlighting the complex regulatory and functional specificity of m^6^A in different cancer types.

### The classification of different patients based on m^6^A-regulated genes is correlated with microenvironment and tissue origin

To explore the impact of m^6^A on tumorigenesis and development in pan-cancer, *m^6^A-express*, the first well-established algorithm to predict condition-specific m^6^A regulation of gene expression from methylated RNA immunoprecipitation sequencing (MeRIP-seq) data [12], was used to screen m^6^A-regulated genes in different cancers. A total of 1527 m^6^A-regulated genes were found in this study (Table S3).

**Figure 4:**
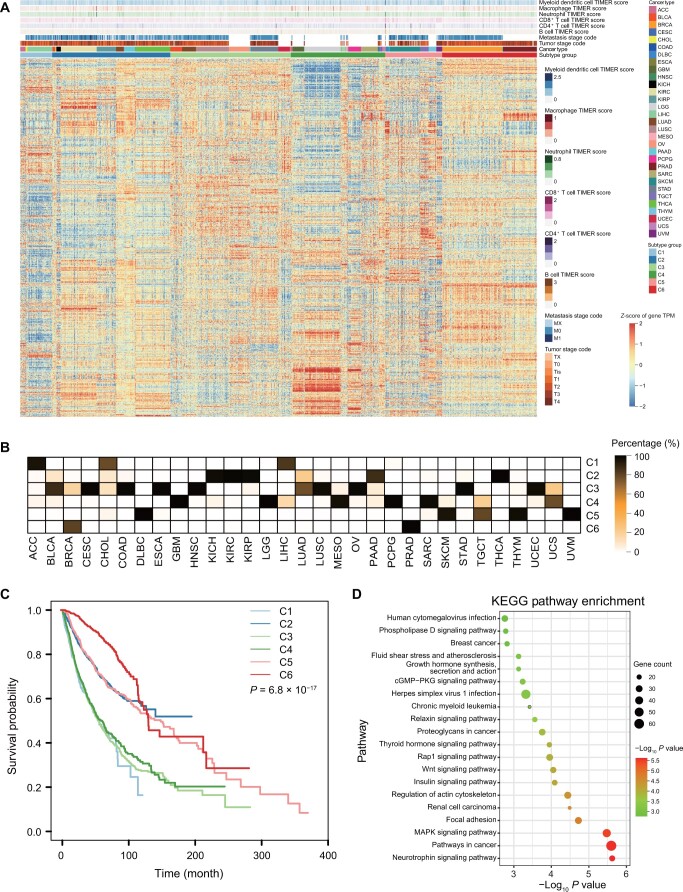
Potential of m^6^A-regulated genes in molecular subtype classification **A**. Heatmap showing the expression of m^6^A-regulated genes of 31 tumors in TCGA. **B**. Percentage of patient numbers in different clusters for each cancer. **C**. Prognostic analysis showing different clinical outcomes of different clusters (*P* = 6.8 × 10^−17^). **D**. KEGG analysis of 1347 m^6^A-regulated genes. TCGA, The Cancer Genome Atlas; KEGG, Kyoto Encyclopedia of Genes and Genomes; TIMER, Tumor Immune Estimation Resource; ACC, adrenocortical carcinoma; BLCA, bladder urothelial carcinoma; BRCA, breast invasive carcinoma; CESC, cervical squamous cell carcinoma and endocervical adenocarcinoma; CHOL, cholangiocarcinoma; COAD, colon adenocarcinoma; DLBC, diffuse large B-cell lymphoma; ESCA, esophageal carcinoma; GBM, glioblastoma multiforme; HNSC, head and neck squamous cell carcinoma; KICH, kidney chromophobe; KIRC, kidney renal clear cell carcinoma; KIRP, kidney renal papillary cell carcinoma; LGG, kidney renal papillary cell carcinoma; LIHC, liver hepatocellular carcinoma; LUAD, lung adenocarcinoma; LUSC, lung squamous cell carcinoma; MESO, mesothelioma; OV, ovarian serous cystadenocarcinoma; PAAD, pancreatic adenocarcinoma; PCPG, pheochromocytoma and paraganglioma; PRAD, prostate adenocarcinoma; SARC, sarcoma; SKCM, skin cutaneous melanoma; STAD, stomach adenocarcinoma; TGCT, testicular germ cell tumor; THCA, thyroid carcinoma; THYM, thymoma; UCEC, uterine corpus endometrial carcinoma; UCS, uterine carcinosarcoma; UVM, uveal melanoma.

These m^6^A-regulated genes were analyzed in 9456 tumor samples from 31 cancers for cluster analysis (Figure S6A and B), and it was found that pan-cancer-related m^6^A-regulated genes could be used for molecular classification and stage classification (**Figure 4**A), indicating that m^6^A contributes to tumor heterogeneity. Interestingly, these different tumor subtypes had different infiltrating levels of immune cells (Figure S7), indicating that m^6^A modifications shape immune responses in the tumor microenvironment and may impact cancer immunotherapy. Furthermore, the classification of these tumors was correlated with tissue origin. For example, kidney chromophobe (KICH), kidney renal clear cell carcinoma (KIRC), and kidney renal papillary cell carcinoma (KIRP), the three different types of kidney cancer, were clustered in C2; two types of lung cancer, lung adenocarcinoma (LUAD) and lung squamous cell carcinoma (LUSC), were clustered in C3; and brain cancer types lower grade glioma (LGG) and glioblastoma multiforme (GBM) were clustered in category C4 (Figure 4B). These m^6^A-regulated genes also had different clinical prognostic values. For example, patients in category C2 had a good prognosis, whereas patients in category C3 with a low infiltrating level of immune cells had a poor prognosis, indicating the influence of m^6^A on the clinical outcome of tumor patients (Figure 4C). It was proposed that m^6^A was a potential target for patients in category C3 who did not respond well to immunotherapy. Consistent with our previous conclusion, these m^6^A-regulated genes were indeed highly enriched in tumor-related pathways (Figure 4D, Figure S8). These results suggest that m^6^A-regulated genes play different roles in diverse cancer types, resulting in distinct clinical relevance and immune status. Moreover, m^6^A may have an impact on the efficacy of immunotherapies. However, the question here is how these cancer-specific m^6^A modifications are established.

### Cancer-specific m^6^A is regulated by cell-specific m^6^A regulators

**Figure 5:**
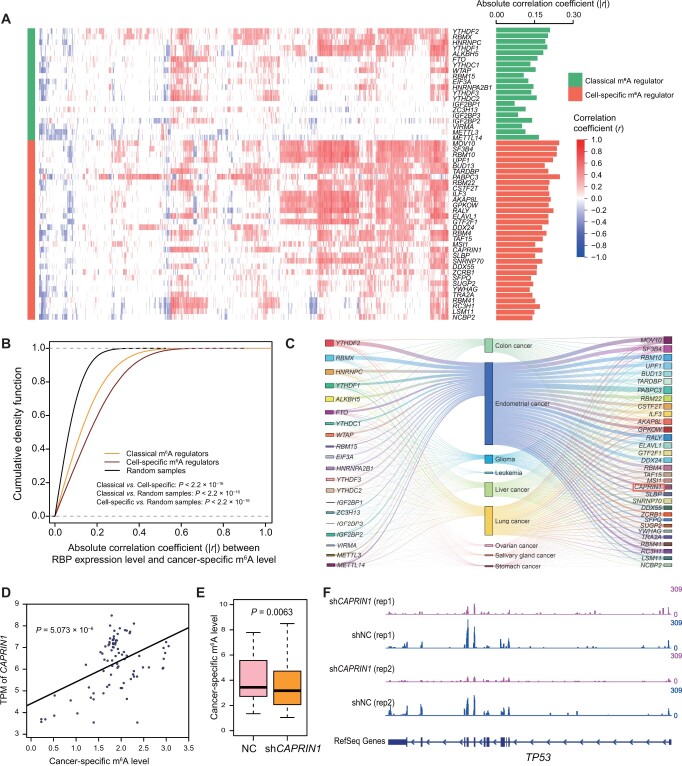
Cell-specific m^6^A regulators are involved in cancer-specific m^6^A regulation **A**. Heatmap showing the correlation between the expression level of m^6^A regulators and corresponding cancer-specific m^6^A level. Positive and negative correlations are indicated in red and blue, respectively. **B**. Plot showing cumulative fraction of absolute correlation coefficient between expression of two types of m^6^A regulator and corresponding cancer-specific m^6^A levels, as well as two types of m^6^A regulator and random cancer-specific m^6^A levels (*P* < 2.2 × 10^−16^). **C**. A Sankey diagram showing the network constructed based on correlation between the expression level of m^6^A regulators and corresponding cancer-specific m^6^A level to identify m^6^A regulators modulating corresponding cancer-specific m^6^A. **D**. Scatter plot showing the correlation between the expression of the *CAPRIN1* gene and the cancer-specific m^6^A level in cancer (*P* = 5.073 × 10^−6^). **E**. Box plot showing cancer-specific m^6^A levels upon *CPARIN1* knockdown (*P* = 0.0063). **F**. Tracks displaying the read coverage of normalized IP input, highlighting the m^6^A levels of the *TP53* gene in the *CPARIN1*-knockdown and control groups. The data range for each track is displayed on the right side (0–309). IP, immunoprecipitation; NC, control group; RBP, RNA-binding protein.

**Figure 6:**
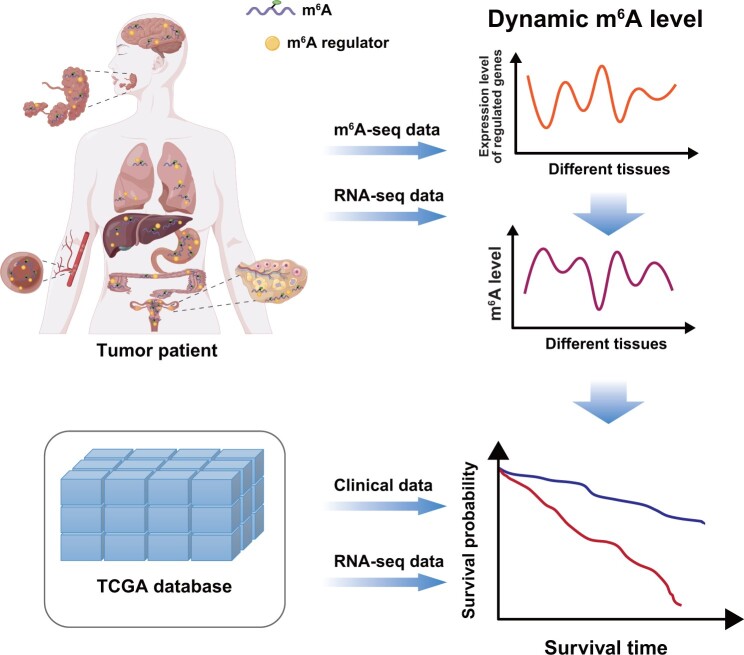
Cancer-specific m^6^A specifically modulated by regulators results in tumor heterogeneity m^6^A-seq, m^6^A sequencing; RNA-seq, RNA sequencing.

Numerous studies have reported that changes in m^6^A, resulting from alterations in the expression of m^6^A regulators, play essential roles in a variety of pathological and physiological processes [21]. We previously identified 32 high-confidence cell-specific m^6^A regulators with a reasonable experimental validation rate that are responsible for global regulation and site-specific m^6^A dynamics through the interplay of classical m^6^A methyltransferases and demethylases at specific sites [21]. Herein, we calculated the Pearson correlations between the level of cancer-specific m^6^A and the expression of each m^6^A regulator. As shown in **Figure 5**A and B, the correlation coefficients between 32 high-confidence m^6^A regulators and cancer-specific m^6^A levels were significantly higher than those of classical m^6^A regulators, indicating that cell-specific m^6^A regulators contribute more to cancer-specific m^6^A levels. We also found that cell-specific m^6^A regulators had greater CVs than classical m^6^A regulators (Figure S9). We then constructed a regulatory network based on the m^6^A regulators and cancer type-specific m^6^A levels according to correlations between the cancer-specific m^6^A levels and the expression of 32 known m^6^A regulators (Figure 5C). Among the m^6^A regulators in the regulatory network, we found that CAPRIN1, a novel METTL3 co-factor [13], was positively correlated with cancer-specific m^6^A (Figure 5D). To further verify the reliability of our conclusion, we validated the regulation of *CAPRIN1* on cancer-specific m^6^A by knocking down *CAPRIN1*. We found that cancer-specific m^6^A was significantly down-regulated upon *CAPRIN1* knockdown in the m^6^A-seq data (*P* = 0.0063, two-tailed Wilcoxon test) (Figure 5E), indicating that *CAPRIN1* regulates the installation of these cancer-specific m^6^A. We also showed an example involving *TP53*, where m^6^A levels of cancer-related genes were significantly reduced in HepG2 cells with *CAPRIN1* knockdown (Figure 5F). p53 expression status is highly associated with cancer-specific survival [22], and the CAPRIN1–m^6^A–*TP53* axis enhances our understanding of p53-based cancer therapies. These results indicate that cancer-specific m^6^A is specifically modulated by cell-specific regulators, leading to tumor heterogeneity and influencing clinical outcomes (**Figure 6**).

## Discussion

In study, we demonstrated the tumor heterogeneity of m^6^A and m^6^A-regulated genes, which contribute to different functions and pathway enrichments. These cancer-specific m^6^A levels and functions are mainly regulated by cell-specific m^6^A regulators.

As a promising therapeutic target, m^6^A is widely involved in various biological processes in tumors, including tumorigenesis, tumor cell proliferation, apoptosis, and drug resistance [21]. For instance, METTL3 is associated with poor prognosis in hepatocellular carcinoma (HCC) patients and promotes HCC cell proliferation through YTHDF2-mediated silencing of *SOCS2* transcription [23]. METTL14 causes the occurrence and development of leukemia by modifying MYB/MYC-targeted genes with m^6^A RNA, and m^6^A promotes the translation of *c-MYC*, *BCL2*, and *PTEN* in leukemia patients [24]. It is widely accepted that tumors exhibit heterogeneity, which influences tumor survival and response to therapy. The detailed regulatory mechanisms of m^6^A in different tumors, particularly whether the effects of m^6^A in one tumor type are applicable to others, remain unclear. Herein, we systematically characterized the depth and breadth of the contribution of m^6^A to interpatient tumor heterogeneity. We also systematically demonstrated downstream pan-cancer-wide m^6^A-regulated genes and upstream comprehensive cancer-specific m^6^A regulators in pan-cancer, promoting our understanding of the mechanisms underlying tumor heterogeneity and the role of m^6^A in tumors. We propose the following suggestions for future studies on the role of m^6^A in cancer precision therapy: (1) the functions and regulatory mechanisms of m^6^A may vary across different cancer types; (2) small-molecule inhibition of m^6^A regulators, such as STM2457 [25], as a strategy against myeloid leukemia, may not be effective for other solid cancers. Each tumor type has its own m^6^A therapeutic targets and regulator inhibitors.

m^6^A regulators play an oncogenic role in different cancer types by targeting essential cancer-related genes [10]. A large number of studies of m^6^A regulatory mechanisms have investigated classical m^6^A regulators, such as METTL3, METTL14, WTAP, METTL16, FTO, ALKBH5, YTH family proteins, and IGF2BPs [26–28]; anti-cancer target drugs targeting METTL3 and FTO have been proven to be effective against cancer [25,29]. However, previous studies have shown that the m^6^A-dependent mechanism cannot be well explained by these 20 m^6^A regulators [12,13]. In fact, we identified hundreds of novel high-confidence m^6^A regulators that were highly associated with m^6^A in different tumor types, indicating a complex regulatory system for m^6^A in tumors. This also highlighted drug targets for m^6^A in addition to the 20 m^6^A regulators. Although previous studies performed pan-cancer analysis based on the 20 classical m^6^A regulator expression profiles, several key questions remained unanswered [14,15]. (1) Changes in gene expression levels do not fully reflect changes in m^6^A levels. (2) m^6^A regulators such as METTL16 function independently of m^6^A to facilitate tumorigenesis, and the effects of m^6^A in cancer may not necessarily be attributable to the effects of m^6^A regulator expression [18]. There are many more m^6^A regulators that function in cancer in addition to the 20 m^6^A regulators previously described [13,26,27,30]. Our discoveries deepen the understanding of the role of m^6^A regulators based on accurate and reliable regulatory networks.

Beyond *trans-*regulation, we believe that *cis-*regulatory mechanisms also play a significant role in m^6^A. Consequently, mutations at m^6^A sites can lead to changes in m^6^A levels, which in turn affect the expression of downstream genes, further influencing the mechanisms. Researchers have developed a series of databases that explore the interplay between m^6^A and genetic variations, such as RMVar, RMDisease, and m^6^A-TSHub [31–33]. In the future, we will integrate these databases to further investigate the landscape and mechanisms of *cis-*regulation in m^6^A. Although many clinical features, especially immune dysfunction, are associated with cancer progression, m^6^A is considered a key regulator of the immune system [34,35]. Due to the limited availability of large samples with both m^6^A-seq and clinical data, it is challenging to investigate the reliable relationship between m^6^A and clinical features. *m^6^A-express* enables the prediction of whole-transcriptome m^6^A regulation of gene expression from m^6^A-seq data in The Cancer Genome Atlas (TCGA). Therefore, we correlated m^6^A with clinical features, including immunological characteristics, in this study. We also identified well-known transcription factors, such as JUN and STAT3, which were found to be targeted and regulated by m^6^A during tumor progression and tumor immunity [36,37]. These factors may contribute to interpatient tumor heterogeneity and impact the effectiveness of immunotherapy, resulting in clinical challenges. Our findings regarding cell-specific m^6^A regulators modulating cancer-specific m^6^A, resulting in dysfunction of the tumor immune microenvironment, are helpful for our further understanding of cancer immunotherapy. It was proposed that immunotherapy combined with m^6^A regulator inhibitors could enhance the efficacy of immunotherapy. However, the detailed regulatory mechanism of m^6^A and the tumor immune microenvironment will require further experimental validation.

## Conclusion

In summary, our study has demonstrated the tumor heterogeneity in m^6^A and m^6^A-regulated genes, which contribute to different functions and pathway enrichments. These cancer-specific m^6^A levels and functions are predominantly regulated by cell-specific m^6^A regulators, resulting in tumor heterogeneity and tumor microenvironment status heterogeneity (Figure 6). Our research not only provides a landscape of the m^6^A profile in different cancer types compared to normal tissues, but also explains the clinical relevance of these specific m^6^A modifications and how these specific regulations are established. To the best of our knowledge, this is the first study based on a large number of m^6^A methylome data to propose that immunotherapy combined with m^6^A modulator inhibitors may enhance the efficacy of immunotherapy. These findings deepen our understanding of the m^6^A regulatory mechanisms in different cancer types and enhance the clinical application of m^6^A across all cancer types.

## Materials and methods

### Data collection and processing of the m^6^A-seq data in multiple tissues

Overall, 93 raw sequence datasets of m^6^A-seq libraries (IP and input) were downloaded from the Gene Expression Omnibus (GEO, https://www.ncbi.nlm.nih.gov/geo/), and four additional m^6^A-seq datasets were generated in this study by collecting samples from the First Affiliated Hospital of Guangxi Medical University, China. These 97 tissue samples were collected from nine tumor types from brain tissue, lung tissue, liver tissue, endometrium, ovarian, blood, colon, salivary gland, and stomach [38–48] (Table S1). In addition, we used the m^6^A-seq data of cell lines collected in a previous study [13] to verify our results, and these data include seven tumor cell lines (HEC-1-A, HepG2, iSLK, MOLM-13, MonoMac6, MT-4 T-cell, and NB4) and three normal cell lines (MSC, NHDF, and TIME) (Table S2).

We used FastQC (v0.11.9; http://www.bioinformatics.babraham.ac.uk/projects/fastqc/) to assess the sequencing quality, and clean data were mapped to the hg38 human reference genome by HISAT2 (v2.2.1) [49]. Then, StringTie (v1.3.3b) [50] was used for assembly and quantification of transcripts per kilobase of exon model per million mapped reads (TPM) of each annotated gene, which were then normalized by the input library. To identify accurate m^6^A sites in the nine types of tumor and adjacent normal tissues, we improved the winscore method as follows [51,52]. In detail, we performed the search for enriched m^6^A peaks by scanning each gene using sliding windows and calculating an enrichment score for each sliding window, which was modified from the method published earlier by Dominissini and his colleagues [52]. We constructed 100-bp sliding windows with a 50-bp overlap across exon regions and determined the reads per kilobase per million mapped reads (RPKM) for each segment. Then, we designated windows with an enrichment score, or winscore, above 2 as m^6^A peaks within individual samples. To mitigate potential inaccuracies from lowly expressed windows harboring unstable winscores, we incremented each window’s RPKM by one in both IP and input datasets prior to winscore computation, thereby down-weighting windows characterized by low RPKMs. Subsequently, we amalgamated the identified m^6^A peaks [13] across all samples for expansive analysis. We derived the m^6^A ratio of every peak by dividing the IP library’s RPKM by the input library’s RPKM. In subsequent stages of analysis, we relegated m^6^A ratios grounded on base values (the input peak’s RPKM falling below 5) as not available (NA). Peaks designated as NAs in a majority of samples were excluded. Following this, we combined adjacent m^6^A peaks within the same gene and partitioned those extending over five continuous windows (equating 300 bp) into several peaks, each confined to a maximum of five windows.

Because different RNA interruption methods used for immunoprecipitation during preparation of different m^6^A libraries can cause changes in the short sequence signal of the same m^6^A peak, the width and center of the same m^6^A peak might be different. Therefore, we took the maximum m^6^A ratio of the combined m^6^A peaks in each sample as the final m^6^A ratio (IP/input). Differences in activity due to different expression levels of m^6^A methylase and demethylase, as well as technical differences in immunoprecipitation efficiency, also contribute to overall m^6^A differences between samples. This dilutes and alters the signal selectively modulated by m^6^A, so we used quantile standardization, which is used to standardize the ratio of m^6^A combined with peaks in all samples [13].

### Analyses of m^6^A across cancer tissues

To compare the m^6^A peaks between cancer and normal tissues, we used the m^6^A identified in tumor tissues according to the above pipeline. To obtain the percentage of peaks enriched in representative motifs of the nine cancer tissues, HOMER software [53] was used for motif enrichment analysis, with randomly permutated sequences as the background for RNAs (HOMER parameter: line = 1000, size = 200). Distributions of m^6^A peaks were plotted on a mega gene with 10 bins in the 5′ UTR, CDS, and 3′ UTR as previously described [13]. A radar plot was drawn using the “fmsb” package implemented in R. We used bamCoverage to obtain an IP library and generate coverage tracks, with bigWig as the output. The short consecutive counting windows were set as 10 bins, and RPKM were used for normalization. With hg38 as the reference genome and *HSPD1*, *TP53*, and *JUN* as target genes, Integrative Genomics Viewer (IGV) (v2.8.13) was used for read coverage of *TP53* in m^6^A-seq data and *JUN* in 97 randomly selected cancer and normal samples [54]. We performed a *t*-test (two-sided, unpaired, unequal variance) on each m^6^A site in tumor and normal tissues; 428 m^6^A sites (adjusted *P* < 0.05) were differentially methylated between tumor and normal tissues.

We calculated the mean and standard deviation (SD) of m^6^A modification intensity across all samples at each m^6^A site. CV is equal to the SD divided by the mean value of the m^6^A sites. According to our previous report [13], sites with CV > 0.3 in specific cancer types were selected as cancer-specific m^6^A sites.

### GSVA and functional annotation

To investigate the activation status of m^6^A modification patterns in different biological pathways across the nine cancer tissues, GSVA enrichment analysis was performed using the GSVA R package [55], which allows for the differential analysis of various pathways at the level of gene sets. We downloaded the gene set “c2.cp.kegg.v7.5.1.symbols.gmt” from the Molecular Signatures Database (MSigDB; https://www.gsea-msigdb.org/gsea/msigdb/), and an adjusted *P* < 0.05 was considered statistically significant. Functional annotation of m^6^A-related genes was performed using the clusterProfiler R package (FDR < 0.05).

### Identification of cancer type-specific m^6^A

We used a highly predictive and sensitive *m^6^A-express* computing framework based on Bayesian negative binomials [12] to evaluate the impact of m^6^A strength (IP) on the expression level (input) of each gene. With hg38 as the reference genome, tumor and normal tissues were used for analysis of m^6^A-regulated genes (*m^6^A-express* parameter: DM_CUTOFF_TYPE = “pvalue”, num_ctl = 2, diff_peak_pvalue = 0.2, FDR = 0.2, isPairedEnd = FALSE, GENE_ANNO_GTF = gtf, isGTFAnnotationFile = TRUE, DIFF_GENE_cutoff_FDR = 0.2, CUTOFF_TYPE = “FDR”). Finally, 1527 m^6^A-regulated genes were screened by *m^6^A-express*. After removing duplicate genes, 1439 unique genes were considered m^6^A-regulated genes (Table S3).

### Clustering analysis in TCGA dataset

We downloaded gene expression data and clinical data including 31 types of tumors (9456 samples), from the TCGA database. By integrating the gene expression and clinical data in TCGA [excluding acute myeloid leukemia (LAML) and rectum adenocarcinoma (READ) with excessive deletion values], consensus clustering was performed to verify the effect of m^6^A-regulated genes on cancer molecular classification. Consensus clustering is an unsupervised clustering method that can distinguish samples into subtypes based on different histological datasets and allows the discovery of new disease subtypes or comparative analysis of different subtypes. To investigate the regulation of m^6^A modification on downstream gene expression, consensus clustering was performed using the “ConsensusClusterPlus” R package (*k* = 6) [56]. A total of 1347 m^6^A-regulated genes with an average TPM > 5 in TCGA were analyzed using Kyoto Encyclopedia of Genes and Genomes (KEGG) and GO analyses. We performed functional enrichment analysis in the Database for Annotation, Visualization and Integrated Discovery (DAVID) (https://david.ncifcrf.gov/) [57], and took the top 5 items ranked in ascending *P* value order as the results. GO enrichment analysis included cellular component (CC), molecular function (MF), and biological process (BP) terms.

### Sub-motif analysis

For our analysis, we first shuffled the m^6^A sub-motif sequences within all GGACA, AGACU, GGACU, and GAACU m^6^A peaks for a specific sample to determine the expected number of windows containing all sub-motifs. Following this, we computed the quantity of windows that had all sub-motifs. This shuffling process was reiterated 10,000 times, yielding 10,000 expected values. To plot and compare results from different samples, we performed normalization by mean-centering the values.

## Ethical statement

The studies involving human participants were reviewed and approved by the Ethics and Human Subjects Committee of The First Affiliated Hospital of Guangxi Medical University, China (Approval No. 2022-E268-01).

## Supplementary Material

qzae052_Supplementary_Data

## Data Availability

The data generated in this study have been deposited in the Genome Sequence Archive for Human [58] at the National Genomics Data Center, Beijing Institute of Genomics, Chinese Academy of Sciences / China National Center for Bioinformation (GSA-Human: HRA006237), and are publicly accessible at https://ngdc.cncb.ac.cn/gsa-human.

## Code availability

The code is available on GitHub (https://github.com/Ansanqi/Pan-cancer_m6A).

## CRediT author statement

**Yao Lin:** Methodology, Validation, Formal analysis, Investigation, Writing – original draft, Writing – review & editing, Visualization. **Jingyi Li:** Software, Formal analysis, Investigation, Writing – review & editing, Visualization. **Shuaiyi Liang:** Software, Writing – review & editing. **Yaxin Chen:** Resources, Writing – review & editing. **Yueqi Li:** Software, Formal analysis, Visualization. **Yixian Cun:** Writing – review & editing. **Lei Tian:** Resources. **Yuanli Zhou:** Resources. **Yitong Chen:** Resources. **Jiemei Chu:** Validation. **Hubin Chen:** Validation. **Qiang Luo:** Visualization. **Ruili Zheng:** Validation. **Gang Wang:** Investigation. **Hao Liang:** Data curation, Supervision, Project administration. **Ping Cui:** Writing – review & editing, Funding acquisition. **Sanqi An:** Conceptualization, Formal analysis, Data curation, Writing – original draft, Writing – review & editing, Visualization, Supervision, Funding acquisition. All authors have read and approved the final manuscript.

## ORCID

0000-0003-1728-3791 (Yao Lin)

0009-0002-6440-5106 (Jingyi Li)

0000-0001-8099-6159 (Shuaiyi Liang)

0000-0002-5469-7080 (Yaxin Chen)

0000-0002-2640-4906 (Yueqi Li)

0000-0002-3951-942X (Yixian Cun)

0000-0002-5268-5270 (Lei Tian)

0000-0001-8855-489X (Yuanli Zhou)

0000-0002-2577-2897 (Yitong Chen)

0000-0003-2942-0490 (Jiemei Chu)

0009-0003-0216-7138 (Hubin Chen)

0000-0002-3753-7325 (Qiang Luo)

0000-0002-3246-0269 (Ruili Zheng)

0000-0001-7264-2457 (Gang Wang)

0000-0001-7534-5124 (Hao Liang)

0000-0001-7195-2832 (Ping Cui)

0000-0002-3177-213X (Sanqi An)
