# Supplementary material for: Pan-cancer Analysis Reveals m6A Variation and Cell-specific Regulatory Network in Different Cancer Types
Source: Genomics Proteomics Bioinformatics. 2024 Jul 5;22(4):qzae052. doi: 10.1093/gpbjnl/qzae052 (PMC11514823; doi:10.1093/gpbjnl/qzae052)
Supplement: qzae052_Supplementary_Data [file qzae052_supplementary_data.zip › Supplementary Figure 1.pdf]

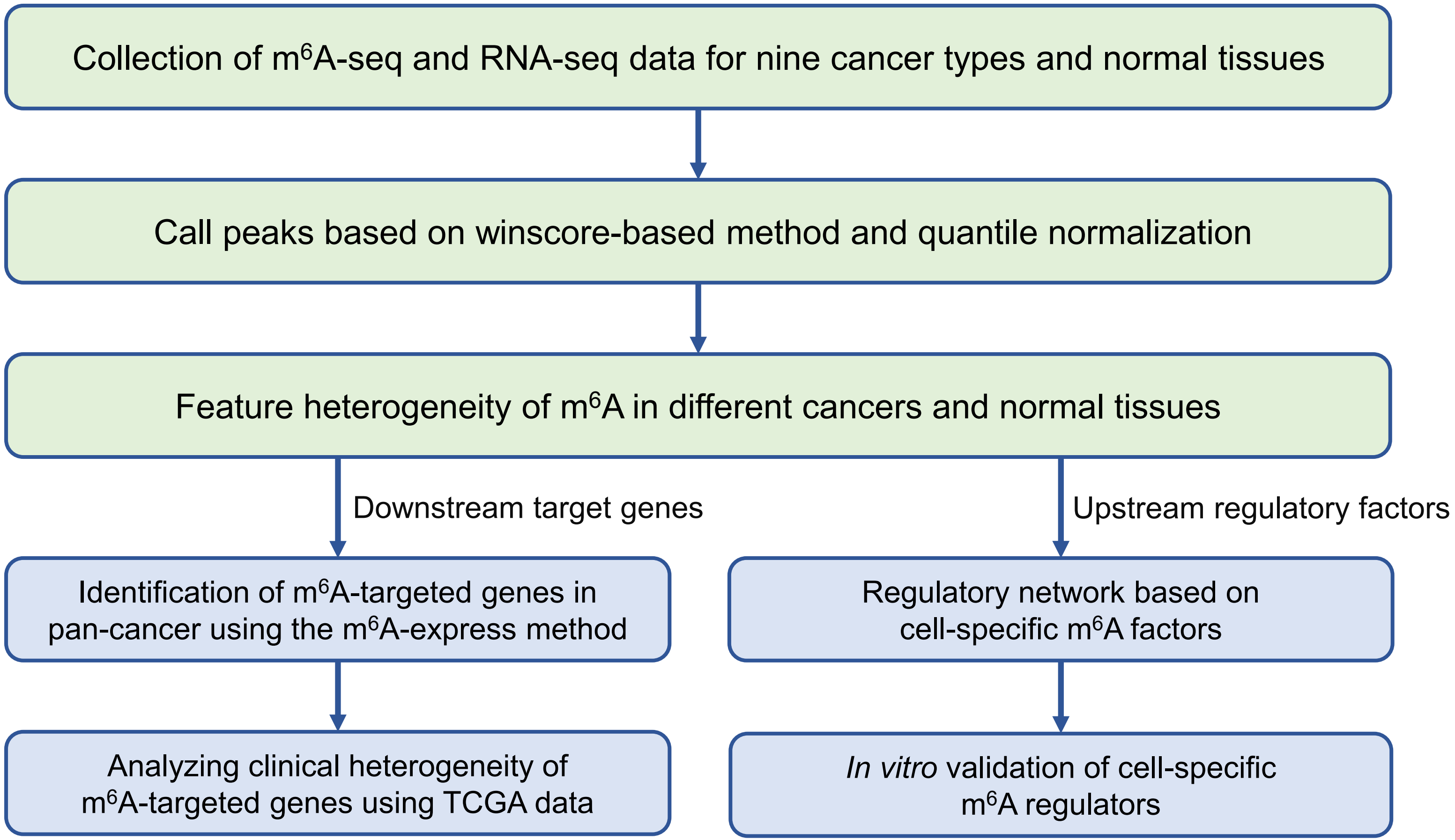
Collection of m6A-seq and RNA-seq data for nine cancer types and normal tissues
Call peaks based on winscore-based method and quantile normalization
Feature heterogeneity of m6A in different cancers and normal tissues
Downstream target genes
Upstream regulatory factors
Identification of m6A-targeted genes in pan-cancer using the m6A-express method
Regulatory network based on cell-specific m6A factors
Analyzing clinical heterogeneity of m6A-targeted genes using TCGA data
In vitro validation of cell-specific m6A regulators
