# Supplementary material for: Pan-cancer Analysis Reveals m6A Variation and Cell-specific Regulatory Network in Different Cancer Types
Source: Genomics Proteomics Bioinformatics. 2024 Jul 5;22(4):qzae052. doi: 10.1093/gpbjnl/qzae052 (PMC11514823; doi:10.1093/gpbjnl/qzae052)
Supplement: qzae052_Supplementary_Data [file qzae052_supplementary_data.zip › Supplementary Figure 2.pdf]

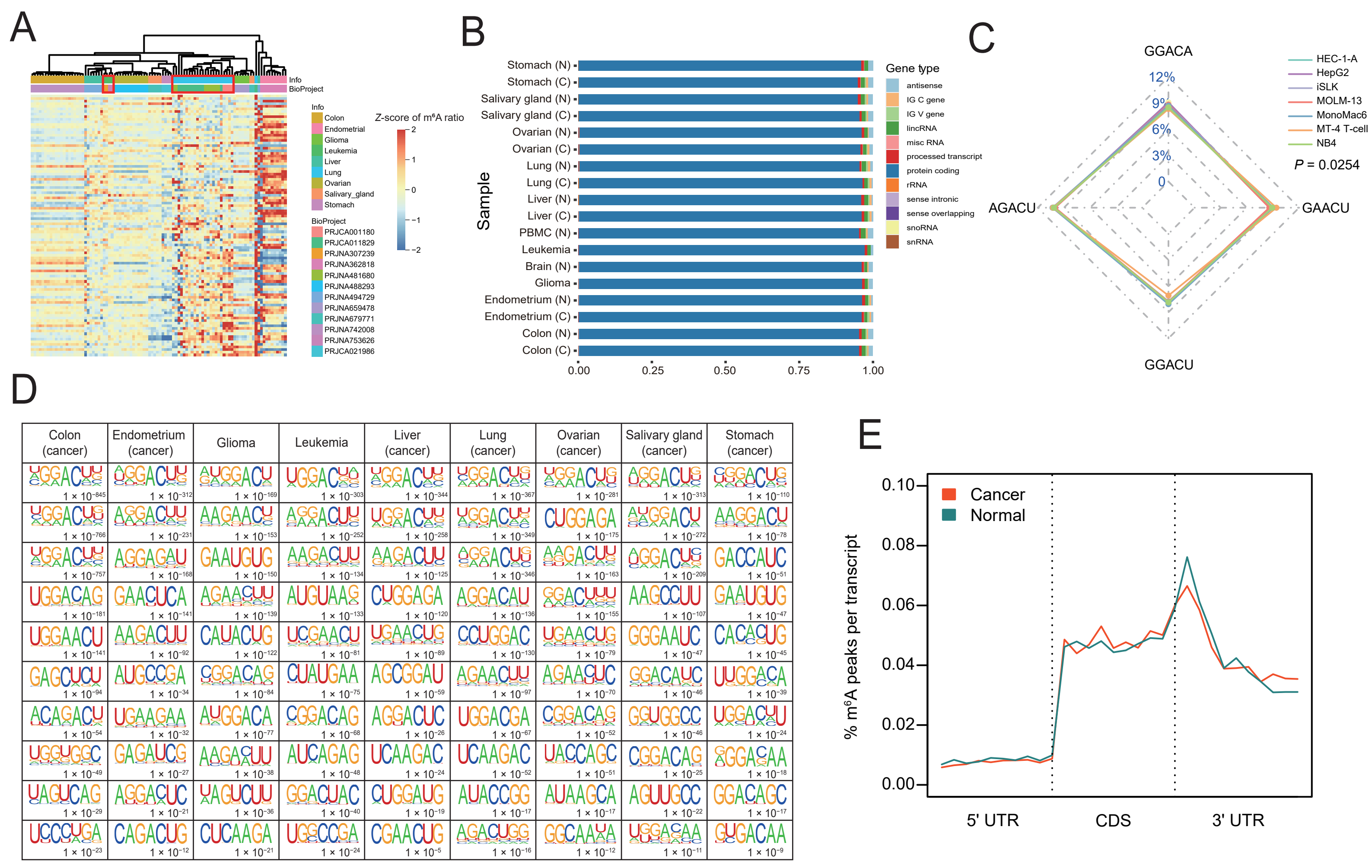
A
Info
BioProject
Info
Colon
Endometrial
Glioma
Leukemia
Liver
Lung
Ovarian
Salivary_gland
Stomach
BioProject
PRJCA001180
PRJCA011829
PRJNA307239
PRJNA362818
PRJNA481680
PRJNA488293
PRJNA494729
PRJNA659478
PRJNA679771
PRJNA742008
PRJNA753626
PRJCA021986
Z-score of m6A ratio
2
1
0
−1
−2
B
Sample
Stomach (N)
Stomach (C)
Salivary gland (N)
Salivary gland (C)
Ovarian (N)
Ovarian (C)
Lung (N)
Lung (C)
Liver (N)
Liver (C)
PBMC (N)
Leukemia
Brain (N)
Glioma
Endometrium (N)
Endometrium (C)
Colon (N)
Colon (C)
0.00
0.25
0.50
0.75
1.00
Gene type
antisense
IG C gene
IG V gene
lincRNA
misc RNA
processed transcript
protein coding
rRNA
sense intronic
sense overlapping
snoRNA
snRNA
C
GGACA
12%
9%
6%
3%
0
AGACU
GAACU
GGACU
HEC-1-A
HepG2
iSLK
MOLM-13
MonoMac6
MT-4 T-cell
NB4
P = 0.0254
D
Colon (cancer)
Endometrium (cancer)
Glioma
Leukemia
Liver (cancer)
Lung (cancer)
Ovarian (cancer)
Salivary gland (cancer)
Stomach (cancer)
E
Cancer
Normal
% m6A peaks per transcript
0.10
0.08
0.06
0.04
0.02
0.00
5' UTR
CDS
3' UTR
