# Supplementary material for: Pan-cancer Analysis Reveals m6A Variation and Cell-specific Regulatory Network in Different Cancer Types
Source: Genomics Proteomics Bioinformatics. 2024 Jul 5;22(4):qzae052. doi: 10.1093/gpbjnl/qzae052 (PMC11514823; doi:10.1093/gpbjnl/qzae052)
Supplement: qzae052_Supplementary_Data [file qzae052_supplementary_data.zip › supplementary material captions.docx]

**Supplementary materials**

**Figure S1 Schematic flow chart demonstrating the process of the analysis**

**Figure S2 m^6^A site characteristics**

**A.** The clustering heatmap showing the clustering effect of m^6^A-seq from different BioProject sources to calculate the abundance of m^6^A sites by improved winscore method. **B.** The proportion of different gene types of m^6^A site in nine tumor and normal tissues. **C.** The radar map showing the percentage of classic m^6^A motifs of 7 cancer cell lines, indicated by different colored lines (Chi-squared test, *P* = 0.0254). **D.** Characteristic motif of m^6^A in nine cancer tissues. **E.** Comparison of overall m^6^A peak distribution between tumor (HEC-1-A, HepG2, iSLK, MOLM-13, MonoMac6, MT-4 T-cell, and NB4) and normal (MSC, NHDF, and TIME) cell lines. N, normal; C, cancer; IG C gene, constant chain immunoglobulin gene; IG V gene, variable chain immunoglobulin gene; lincRNA, long intergenic non-coding RNA; misc RNA, miscellaneous RNA; rRNA, ribosomal RNA; snoRNA, small nucleolar RNA; snRNA, small nuclear RNA; UTR, untranslated regions; CDS, coding sequence.

**Figure S3 Variable m^6^A sites characteristics**

**A.** The proportion of stable and variable m^6^A peaks is displayed by a stacked bar chart across all tumor and normal tissues analyzed in this study. **B.** CV of m^6^A level in tumor and normal tissues. The boxplot showing the CV of m^6^A level at the 5' UTR (*, *P* < 0.05; **, *P* < 0.01; ****, *P* < 0.0001). **C.** The track shows the m^6^A coverage of the *HSPD1* gene from randomly selected samples among 97 cancer and normal subjects. The data range for each track is displayed on the right side (0–96). **D.** Differences in m^6^A between cancer and normal samples (*P* < 2.2 × 10^−16^).

**Figure S4 GSVA enrichment analysis revealed activation status of different cancer-specific m^6^A-related KEGG pathways**

After being Z-score normalized, the GSVA sample-wise gene set enrichment scores are used to plot a heatmap. GSVA, gene set variation analysis.

**Figure S5 GO enrichment analysis of cancer type-specific m^6^A across nine cancer types**

The X-axis represents the significance level of pathway enrichment. **A.** Cellular component. **B.** Molecular function. GO, gene ontology.

**Figure S6 Classification of 31 cancer types based on m^6^A-reg-exp gene profiles**

**A.** Consensus clustering matrix of m^6^A-reg-exp genes in 31 tumors in TCGA for k = 6. **B.** Consensus clustering CDF for k = 2 to k = 12. CDF, cumulative distribution function.

**Figure S7 Plot of cumulative fraction and boxplot in 6 subtypes of immune cell**

**A.** B cell. **B.** CD4^+^ T cell. **C.** CD8^+^ T cell. **D.** Macrophage. **E.** Myeloid dendritic cell. **F.** Neutrophil. TIMER score calculated by TIMER. TIMER, Tumor Immune Estimation Resource.

**Figure S8 BP terms of 1347 m6A-reg-exp genes by GO enrichment analysis.**

Bubble size represents the number of enriched genes, and bubble color represents −log_10_ *P.* BP, biological process.

**Figure S9 Heatmap of expression of classical m^6^A regulators and cell-specific m^6^A regulators in 31 tumor samples in TCGA**

Bar graph shows the CV value of corresponding genes. CV, coefficient of variation.

**Table S1 Data collection of 9 types of cancer and normal tissues m^6^A-seq**

**Table S2 Data collection of 7 types of cancer cell lines m^6^A-seq and 3 types of normal cell lines m^6^A-seq**

**Table S3 m^6^A-express result delineates cancer specific genes that are regulated by m^6^A in distinct cancer types, alongside their expression levels across diverse samples**
