# Supplementary figures and images for: Pan-cancer Analysis Reveals m6A Variation and Cell-specific Regulatory Network in Different Cancer Types
Source: Genomics Proteomics Bioinformatics. 2024 Jul 5;22(4):qzae052. doi: 10.1093/gpbjnl/qzae052 (PMC11514823; doi:10.1093/gpbjnl/qzae052)

**A**

Fraction of peaks

0.0 0.2 0.4 0.6 0.8 1.0

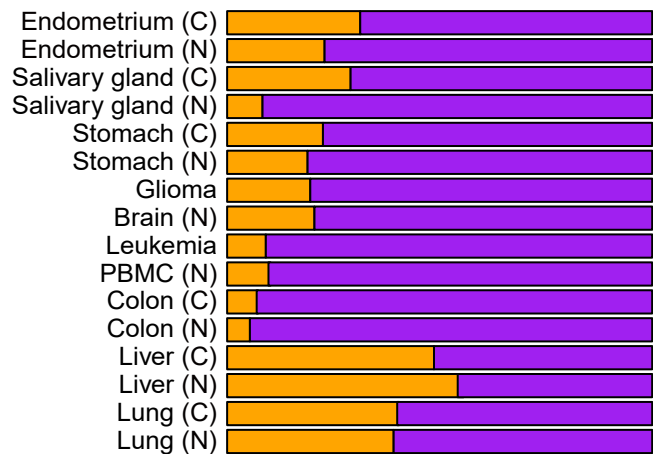

Variable peaks Stable peaks

**B**Group ■ Cancer ■ Normal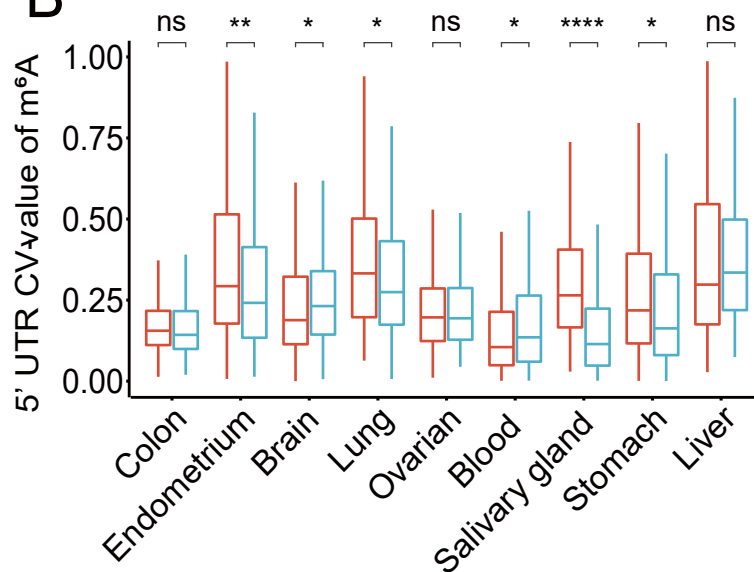**C**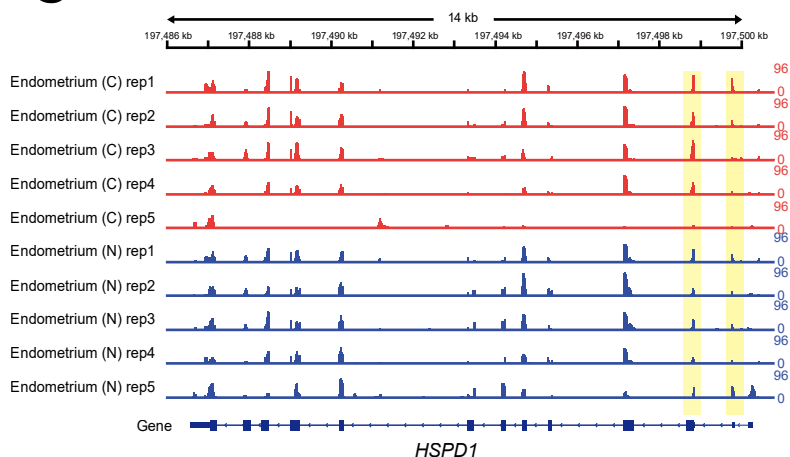**D**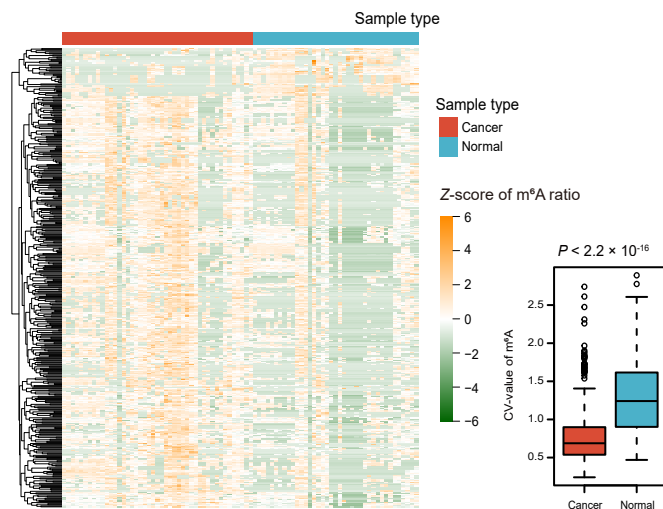

Supplement: qzae052_Supplementary_Data [file qzae052_supplementary_data.zip › Supplementary Figure 3.pdf]

# Cancer type

## Cancer type

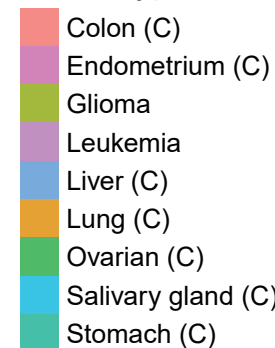

## Z-score of GSVA score

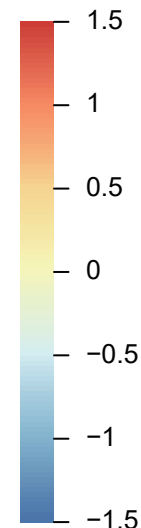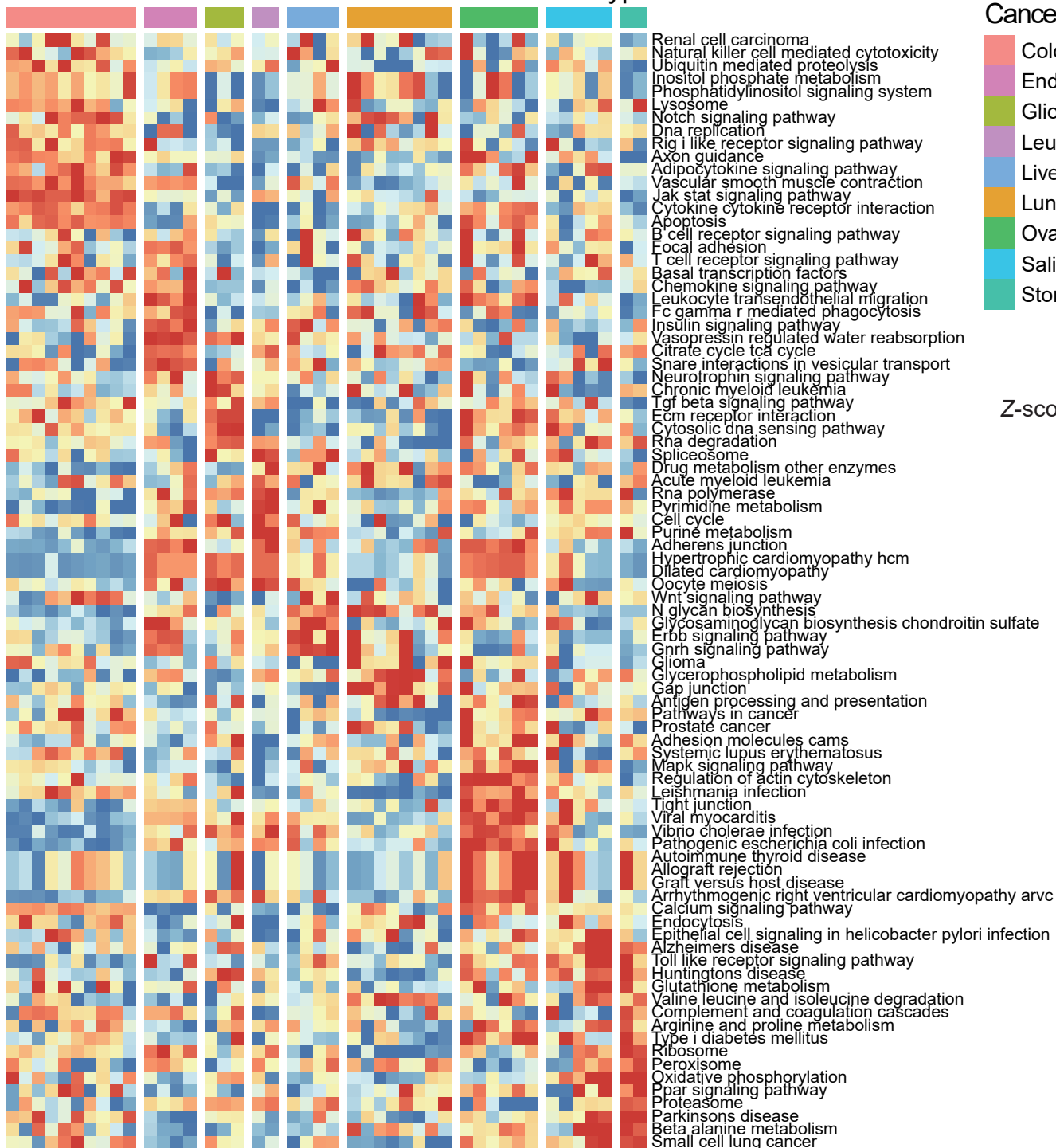

Supplement: qzae052_Supplementary_Data [file qzae052_supplementary_data.zip › Supplementary Figure 4.pdf]

A

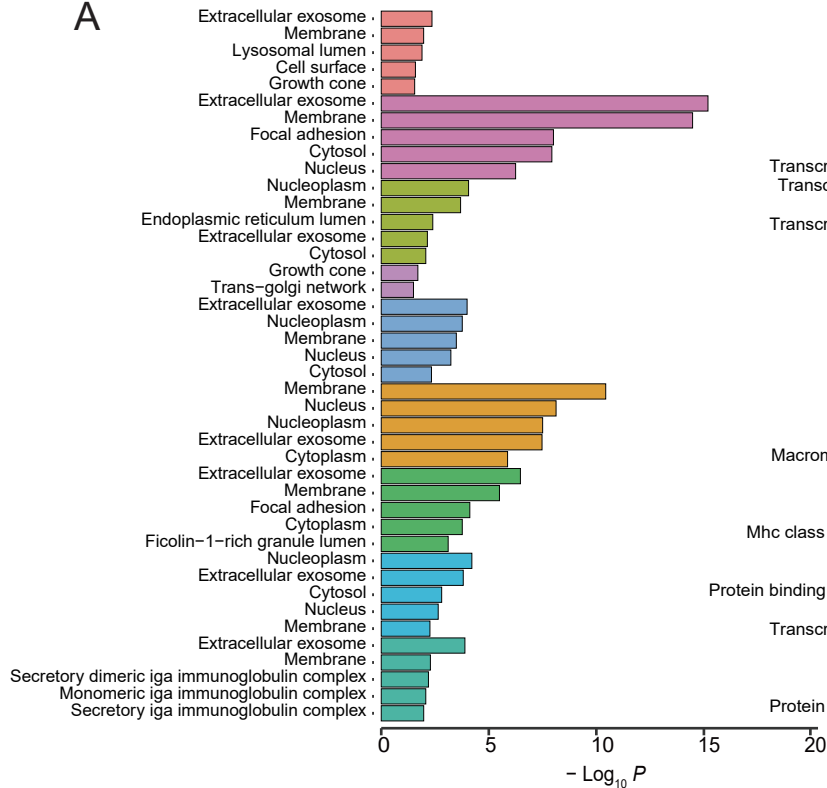

B

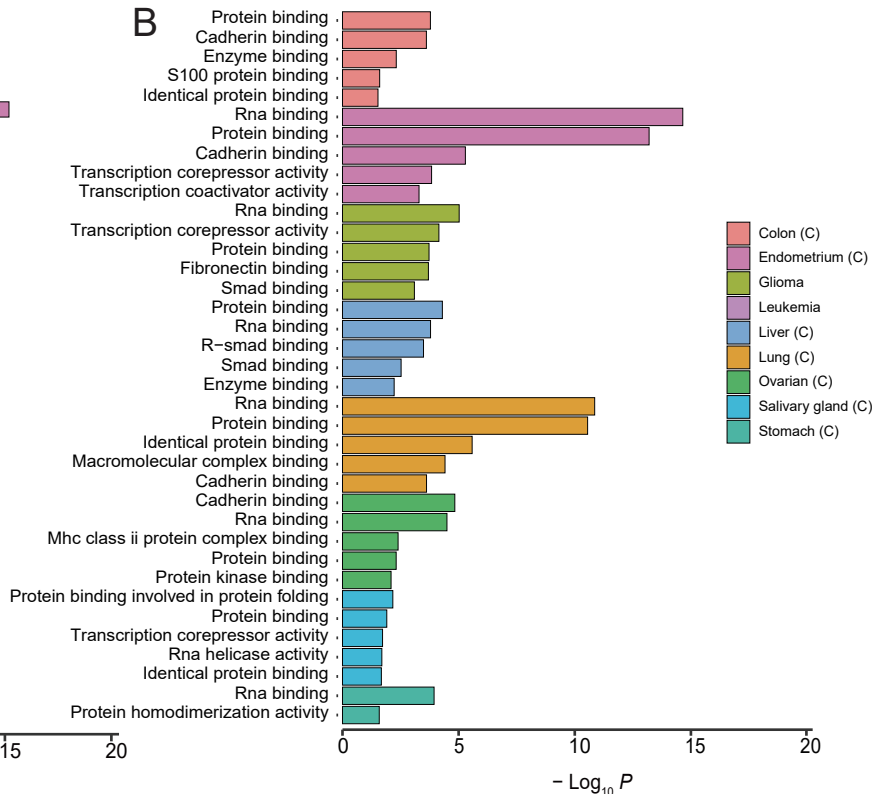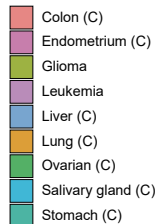

Supplement: qzae052_Supplementary_Data [file qzae052_supplementary_data.zip › Supplementary Figure 5.pdf]

A

Consensus matrix  $k = 6$ 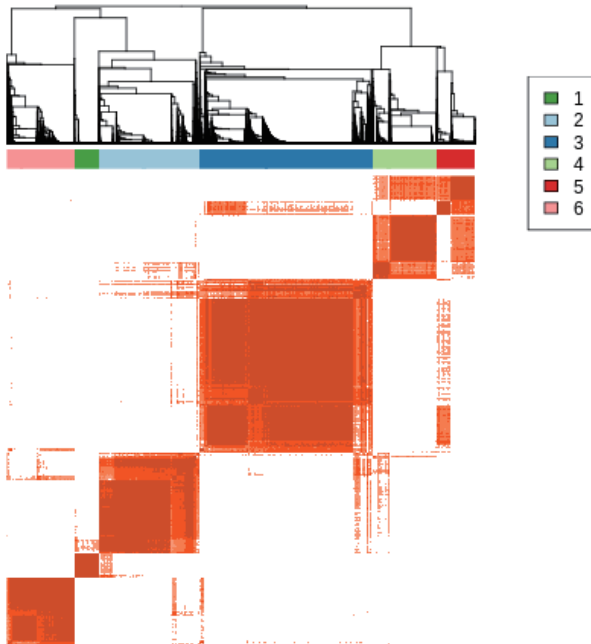

B

Consensus CDF

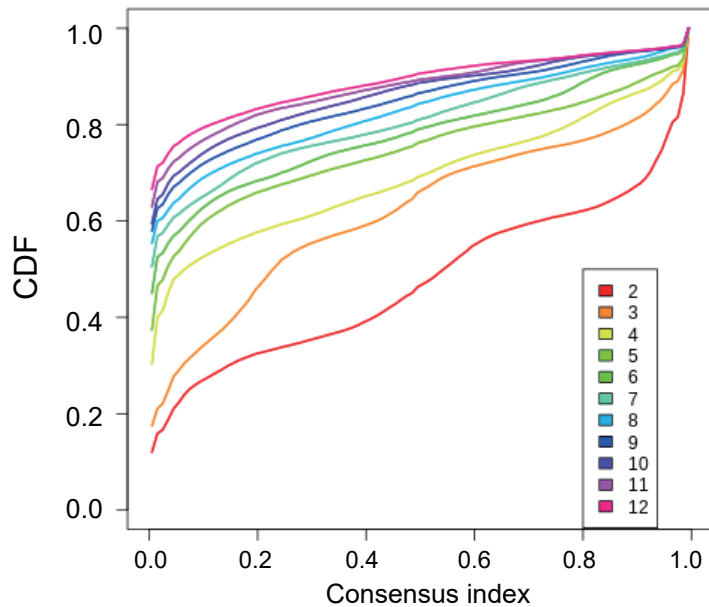

Supplement: qzae052_Supplementary_Data [file qzae052_supplementary_data.zip › Supplementary Figure 6.pdf]

C1 C2 C3 C4 C5 C6

A

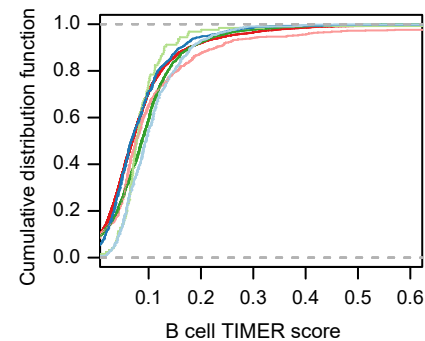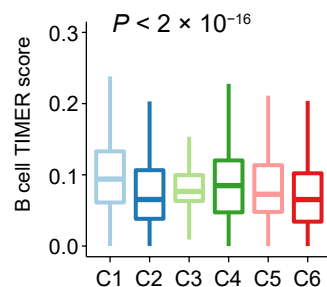

B

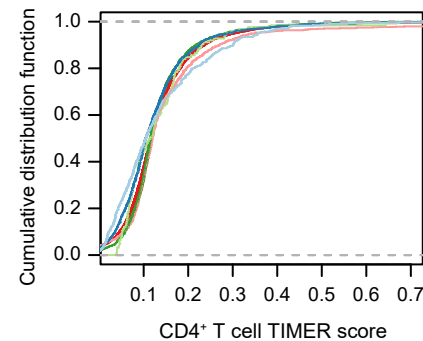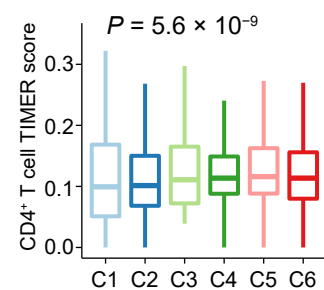

C

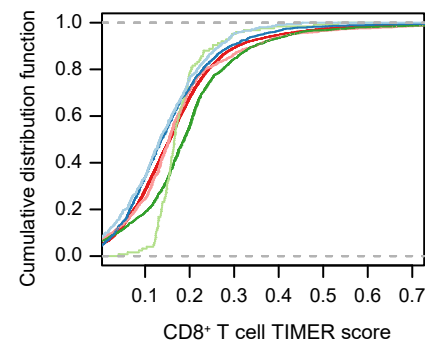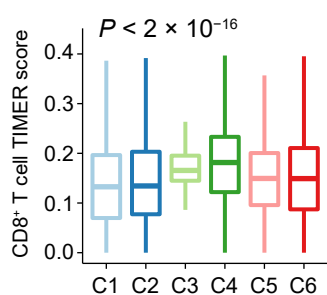

D

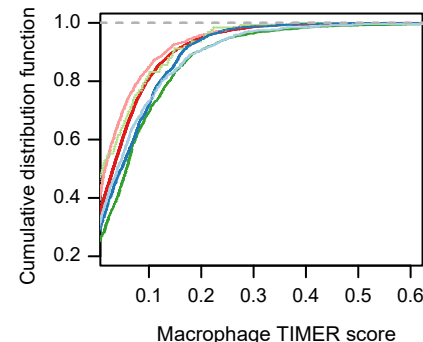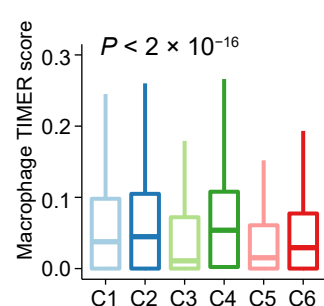

E

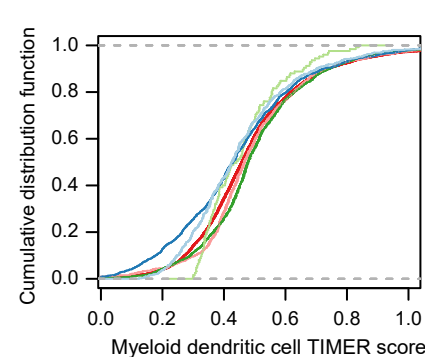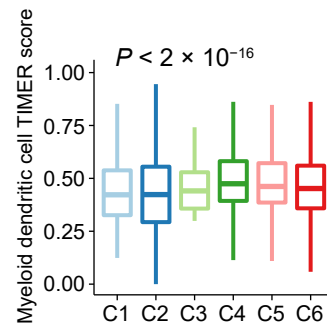

F

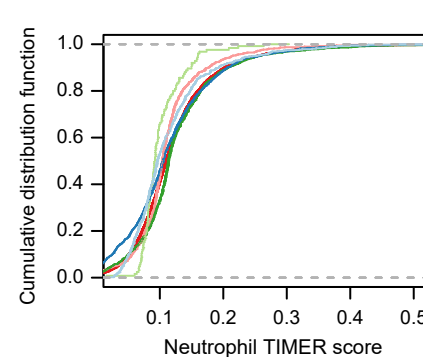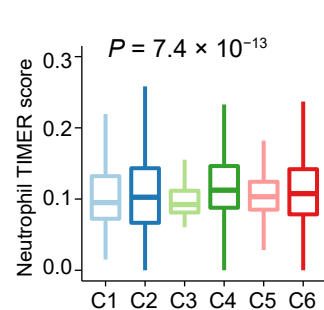

Supplement: qzae052_Supplementary_Data [file qzae052_supplementary_data.zip › Supplementary Figure 7.pdf]

# GO enrichment (BP)

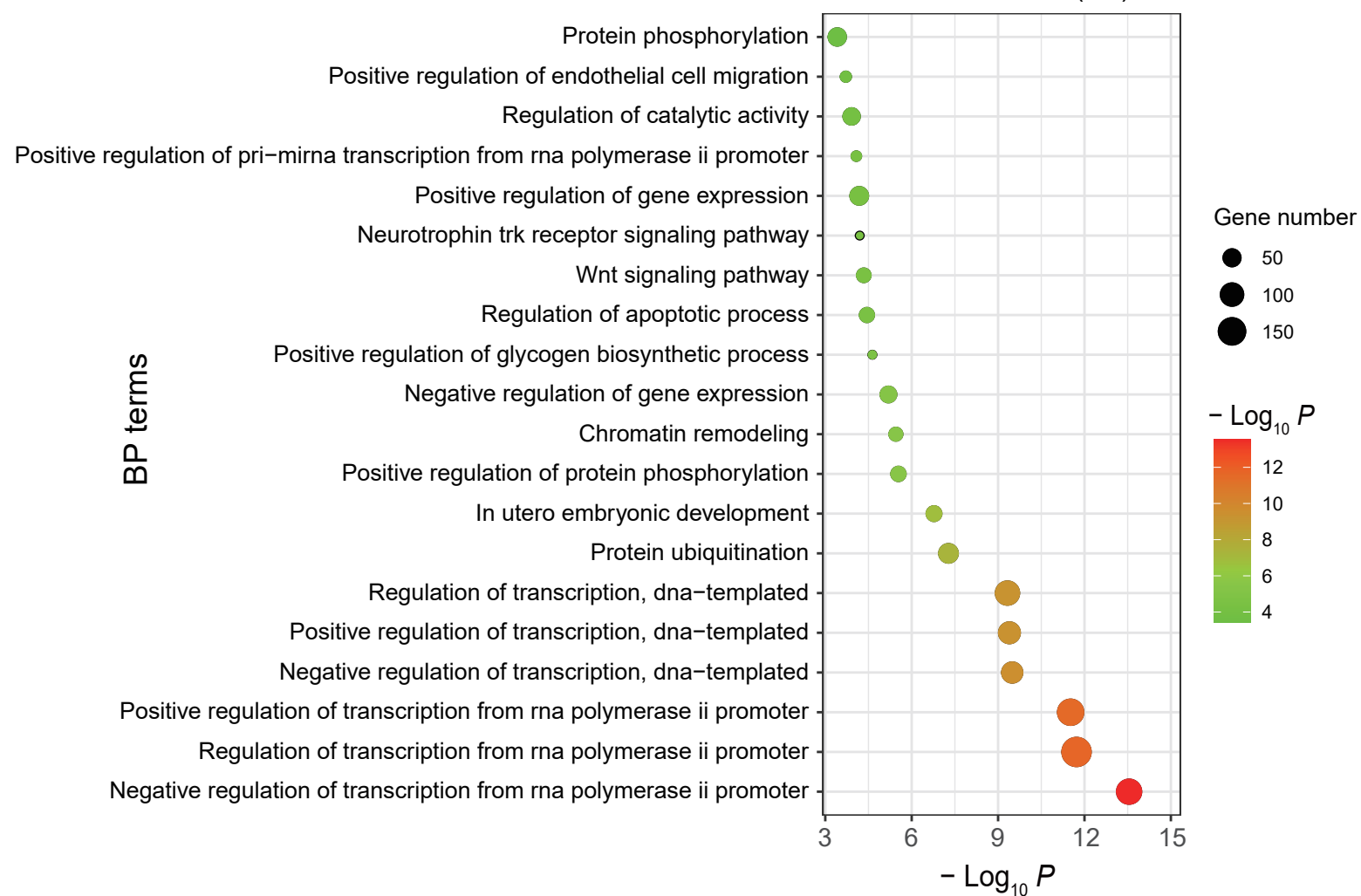

Supplement: qzae052_Supplementary_Data [file qzae052_supplementary_data.zip › Supplementary Figure 8.pdf]

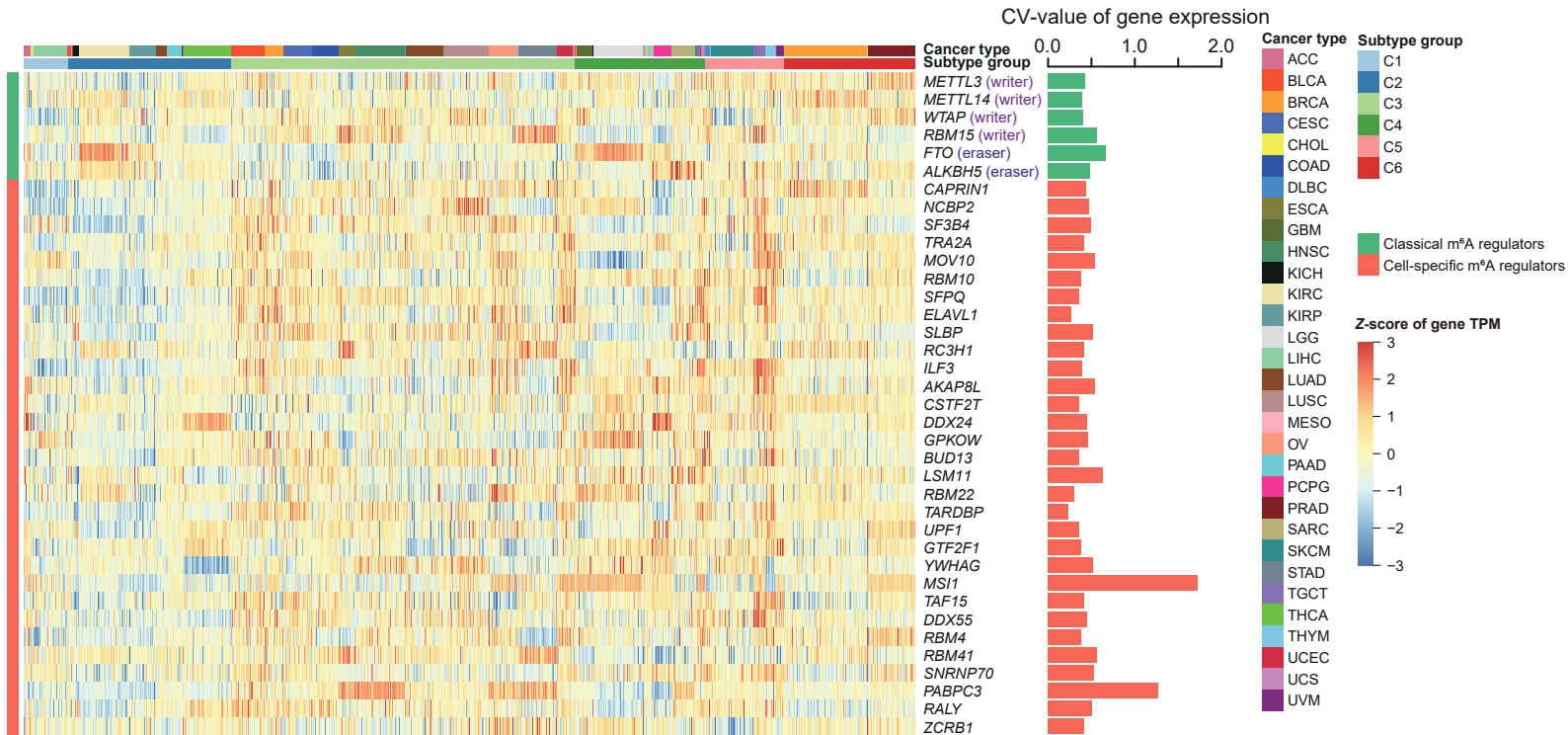

Supplement: qzae052_Supplementary_Data [file qzae052_supplementary_data.zip › Supplementary Figure 9.pdf]
